# W. Kimryn Rathmell receives the 2025 Stanley J. Korsmeyer Award

**DOI:** 10.1172/JCI204431

**Published:** 2026-01-16

The American Society for Clinical Investigation (ASCI) honors W. Kimryn Rathmell, MD, PhD (Figure 1), with the 2025 ASCI/Stanley J. Korsmeyer Award. Dr. Rathmell is recognized for her contributions to understanding the biologies driving cancer arising in the kidney and her advocacy for the careers of physician-scientists. Dr. Rathmell recently became CEO of The Ohio State University (OSU) Comprehensive Cancer Center — Arthur G. James Cancer Hospital and Richard J. Solove Research Institute. She is former director of the National Cancer Institute (NCI), before which she was Hugh Jackson Morgan Professor in the Department of Medicine at Vanderbilt University Medical Center and served on the faculty of the University of North Carolina at Chapel Hill. Dr. Rathmell was elected to the National Academy of Medicine and the American Academy of Arts and Sciences, is a fellow of the American Association for the Advancement of Science, and is a past president of the ASCI (2019–2020). Dr. Benjamin D. Humphreys, MD, PhD, ASCI Past President (2023–2024), Joseph Friedman Professor of Renal Diseases in Medicine, and Chief of the Division of Nephrology at Washington University in St. Louis, interviewed Dr. Rathmell at the AAP/ASCI/APSA Joint Meeting in Chicago in April 2025.

Benjamin D. Humphreys: The Korsmeyer Award is particularly special for the ASCI because it values equally the recipient’s scientific accomplishments as well as their legacy of mentoring, and I look forward to hearing from you on these topics. Please tell us a little bit about yourself before I dive into the questions.

W. Kimryn Rathmell: I’m a physician-scientist focusing my efforts on kidney cancer. That’s a passion project that I’ve been interested in diving into for the last 20–25 years, both scientifically and in terms of improving the lives of patients. I’m most recently the director of the NCI and have spent most of my career in academia working as a medical oncologist with developing physician-scientists as well as a division chief and a department chair working across the healthcare sector. The next step in my career is moving on to be the CEO of The Ohio State James Cancer Hospital.

BDH: Could you tell us a bit about how you came to focus your research on the mechanisms and biology that drive kidney cancers?

WKR: It’s one of those stories that’s a little bit serendipitous. I was always interested in how normal cells can go awry. My PhD work was studying DNA repair, how normal mechanisms that keep your cells safe get switched into causing cells to become cancerous or damaged in other ways. When I became a fellow, I was looking for some similar opportunity and area of interest. And it was just at that time that how hypoxia signaling and how our cells respond and sense levels of oxygen and enact programs that can survive a period of deprivation that actually usurp cancer were discovered. And kidney cancer happens to be the hallmark cancer [in terms of the involvement of hypoxia]. This is a mechanism that’s relevant for many cancers, but in kidney cancer, there’s a hardwired switch that turns on the hypoxia program. This normal response is a perfect storm: turning on changes in metabolism, in angiogenesis, and in how cells choose to survive or die and migrate. I just found that a fascinating area to dive into. Other people did, too; the Nobel Prize was awarded for this discovery. I was a part of that cheering squad. At the same time, I was looking at how to become relevant in the clinical space — seeing a lot of patients with kidney cancer and with nothing to be offered. High-dose IL-2 was approved but very rarely given. The likelihood of it having an effect was minimal. Most patients were referred directly to hospice. That was my role as a doctor. So it seemed like a rich opportunity for the science to drive improvement. That played out for many years, with quite a separation between trying to make advances in the clinic and what was happening in the laboratory. But they’ve gradually become much closer. Today, kidney cancer is a completely different story. The ways that kidney cancer can be treated, even cured: it’s just phenomenal. It’s the dream completely realized.

BDH: It sounds courageous. But if there were so few treatments at the time, did you worry that it was risky to go into this field at all?

WKR: It was risky only in that there was no one else in the field. I distinctly remember several conversations where I said, “I’m going to be a kidney cancer doctor,” and being told, “You won’t have enough volume of patients; there’s no community to be a part of. You have to be a genitourinary cancer doctor.” And that’s what I essentially was. I saw a lot of prostate and bladder and testicular cancer, but also all the kidney cancers. As the therapies started to come, and suddenly patients were living longer, then my clinic shifted because there were kidney cancers to see. So it was much less risky than I thought it would be. But I wasn’t terribly worried about the risk, because as a physician-scientist, my major focus was in the laboratory, and there was so much to do there.

BDH: What factors do you feel have been most critical to your success in the laboratory?

WKR: I think the success that we have almost always comes down to the people you work with. The laboratory is an ecosystem of students, postdoctoral fellows, and other scientists and collaborators. It’s having people who challenge you to think creatively. We’ve gone in lots of different directions. We sometimes have described the laboratory as the “blind men and the elephant.” We have studied everything from angiogenesis and chromatin biology to tumor microenvironment and transcriptomics, based on the talents of the individuals, the interests that they had, or what was ripe at the time. It was the people that drove it. But I think the other piece is that we were always closely tied to the clinic. Even though we were not a traditionally translational laboratory where everything is clinic-focused, we went out of our way to try to move as quickly as we could into questions that we could address in patients. Whether using imaging modalities or studying tissues, this added to the relevance and often took our experiments in different directions that we might not have expected had we stayed in the model systems. It was this combination that helped us to find some of the answers that have been the most surprising and impactful.

BDH: I’m sure the science took you to all these different areas in cancer biology, but also perhaps matching the talents and personalities of your trainees with the work drove progress as well.

WKR: That is a story we can tell over and over again. People come in, and they’re fascinated by some aspect. As a mentor, you have to help guide them. It has to fit. We have to have funding for it. It’s not a free-rein candy store. But it’s trying to marry the science to what gets them passionate. I’ve also seen repeatedly people who appear somehow not to have the drive or passion, but once they found something that they were interested in, then they’re on their way. Because then it’s fun.

BDH: Where do you see your work going in the future?

WKR: We could go in a lot of potential different directions. Number one, it will depend a lot on who the lab is composed of. I mentioned [the move to OSU], and it depends who comes. I could easily see us staying in metabolism to understand how we could influence this rich area to change the way the immune microenvironment interacts with tumors. I’m not an immunologist, but [immunology] is an area that I would go to if I had a scientist who wanted to work with me and who comes with a strong enough background to really dive in. That’s where the gold mine has been. That’s where we’re starting to see real cures achieved. When I was at the NCI, we got much more interested in the rare subtypes. When we say kidney cancer, we mostly mean clear cell renal cell carcinoma. That’s 75% of tumors, but 25% is a whole laundry basket of 20-some different subtypes. The knowledge [about these rare subtypes] is where we were 20 years ago [with clear cell renal cell carcinoma]. I would love to, with the luxury of more experience and grounding, be able to take on one or two of those rare subtypes and help them get to the place where clear cell is.

BDH: I want to switch to mentoring and ask you how have you approached mentorship in your lab and during your time as department chair and in your other leadership roles?

WKR: I think of them very similarly, and it’s nice the way the career arc works, from learning how to run a laboratory to learning how to run a bigger team, learning how to run a division to a department, to [directing] the NCI. In the laboratory, as I mentioned, it’s working with someone to help find what is most exciting to them. We usually start with a project and allow it to percolate for a bit, then define it more a few months down the road. I think there has to be an element of patience. That’s also true at some of the higher levels of mentorship. For me, it’s important to know the team very well, for us to be able to be candid or transparent or honest with each other so that we can — with a little time and probing — know where people’s sweet spots are and where they can have the greatest impact. I like to run a team that allows everyone the opportunity to be able to bring things up: a collaborative style of leadership that probably comes from how I run the laboratory. That’s how I operate.

BDH: Do you feel like that team building is important so that your trainees can feel comfortable challenging each other and making ideas incrementally better?

WKR: A hundred percent. The thing I love about science and medicine is that we embrace, or we should [embrace, situations where] a laboratory member challenges a thought or idea. You have the opportunity to find out that there’s something that you didn’t expect at all. And that’s the most fun. You don’t get that if you just say, “We were right!” You get much better answers [with the mindset] “I think that’s wrong. What else could it be?”

I was lucky that one of my very early students, my first student, was able to show how to push that. We had made a mouse model and were just getting started. Mouse number 5: I still remember this. We have so many slides from mouse 5, and it had lots of cancers. We thought, “We have hit the jackpot here. We just have to [analyze] a few more, and we’ll know.” And she rigorously did the numbers, went through every kidney, every tumor of hundreds of mice to prove that we were wrong. But that helped us see that there was some other very interesting biology to understand. Had we not had her willingness to speak up at an early stage in the laboratory, the culture might not have been set up that it’s very okay to speak up. Also, the people who are working right there in the spaces, they see what’s happening. I want them to be able to say, “I’m seeing something unusual under the microscope.” Sure, it’s possible that it’s some artifact, and we need to fix the scope. But maybe they’re seeing something that’s completely new. And we’ve encountered that as well.

The other story that I’ll tell [relates to] an area that I’d love the laboratory to keep going in, but it would take the right person to join us so we could keep collaborating. I had a scientist in the laboratory who was a really good cell biologist, and we were looking at DNA and at chromatin patterns and cellular division. He observed that there appeared to be chromosomes with two centromeres, which is wrong. They are only supposed to have one. At first you think, “That’s an anomaly. There are a million reasons why it’s not real.” He then developed the technologies that we needed to be able to interrogate that. It turns out, in fact, [that such cell divisions] happen and at a level that’s not insignificant. Once you see it, you can’t unsee it. But we’d never seen it for the first many years that we were looking at these unusual cell divisions. We were seeing breaks and chromosomal bridges and not having a clue why they were there. It was his discerning eye that completely changed the direction of the lab. I’d love to continue to collaborate with him. He has his own lab now. So that work continues.

BDH: We’re in a remarkable time in biomedicine, and the physician-scientist career path is so rewarding. We’re also in 2025, and it’s a challenging time for academic medicine. How do we best encourage our early-career physician-scientists to continue on this wonderful path?

WKR: I think that’s the important question, because it is an incredibly valuable place to work, between patient care and discovery science. We are not mechanics of the human body; we are inventing the space for health. When it’s so challenging around funding climates and other external pressures, it can be easy to choose alternative careers, so I very much worry about our workforce. My encouragement to people who are interested in this field is: We need improvements for human health. There is a lot left to discover. We’re at a place where the opportunities to make those impactful discoveries happen much, much faster, in ways that can change the course of diseases not in lifetimes, but even in time that you might measure in months or years. Who would not want to be a part of that? Physician-scientists have always been here, and through the challenges that are ahead — mirroring those that have been behind us — this is still a field that will be here. I really encourage people to stick with it, find good mentors, find people who will support them, find teams where they can do the work that they want to do, and be a little flexible in how things happen. Even in the best of scenarios, this [career path] is not a menu of things. It involves a lot of being willing to think differently and having someone challenge your ideas. That is a part of it. Accepting some flexibility gives us resilience to work in this fascinating space.

BDH: Dr. Rathmell, congratulations again on being the 2025 recipient of the Korsmeyer Award, and thank you for sharing your inspiring stories.

WKR: Thank you very much.

*The interview has been edited for length and clarity*.

## Figures and Tables

**Figure 1:**
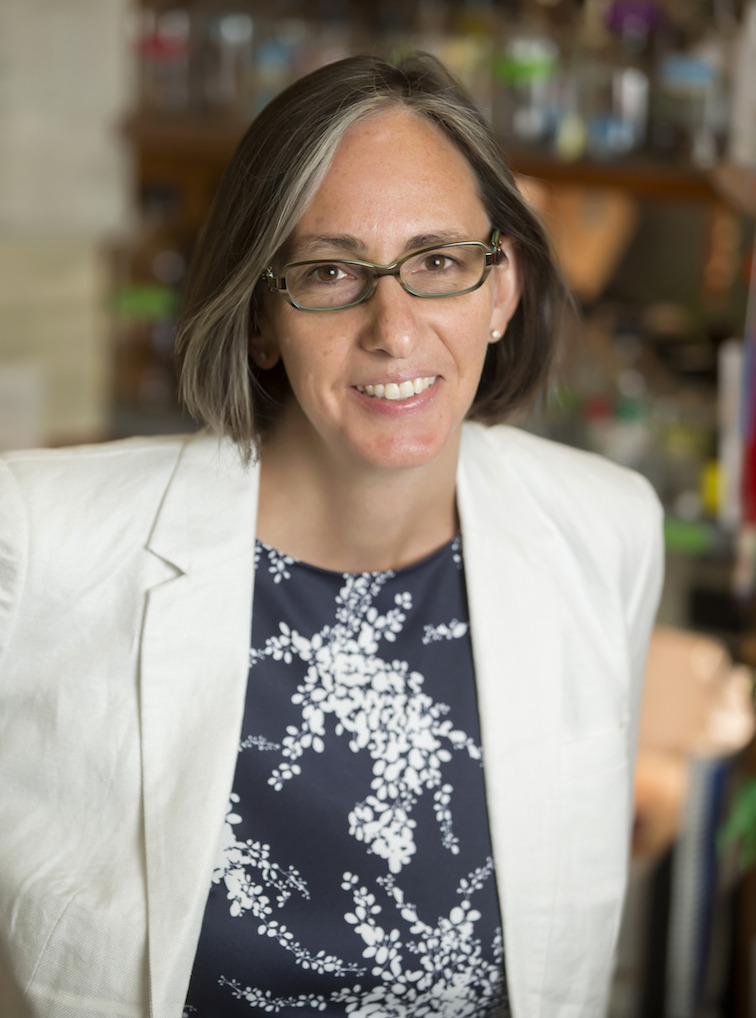
W. Kimryn Rathmell is the recipient of the 2025 Stanley J. Korsmeyer Award.

## Version 1 01/16/2026

Electronic publication

